# Bringing Policymakers to Science Through Communication: A Perspective From Latin America

**DOI:** 10.3389/frma.2021.654191

**Published:** 2021-04-26

**Authors:** Marta Pulido-Salgado, Fátima Antonethe Castaneda Mena

**Affiliations:** ^1^Investigación y Ciencia (Spanish Edition of Scientific American, Springer Nature), Barcelona, Spain; ^2^UNESCO Chair Con-E-Ect, Guatemala, Guatemala

**Keywords:** science communication, science advice, Latin America, science diplomacy, evidence-base for policy

## Abstract

Scientific knowledge should be shared beyond academic circles in order to promote science in policymaking. Science communication increases the understanding of how the natural world works and the capacity to make informed decisions. However, not every researcher has the ability to master the art of communicating, and even less in a clear, concise, and easy to understand language that society representatives appreciate. Within the huge and extraordinarily diverse Latin American region, science communication has been going on for at least 200 years, when the first science stories appeared in the newspapers, as well as the first science museums and botanical gardens were founded. Nevertheless, resources are limited, and notably time, which researchers spend mostly in mentoring, ensuring funding, publication of their results and laboratory work, while science journalists are an endangered species. This perspective article aims at providing some recommendations to build bridges between science and decision-making parties through communication, by exploring how Latin American diplomats and policymakers engage with scientific knowledge.

## Introduction

In its 27th Article, the Universal Declaration of Human Rights states that “Everyone has the right freely […] to share in scientific advancement and its benefits.” Scientific knowledge empowers citizens by increasing their capacity to make informed decisions and strengthens democracy by promoting debate. Understanding the scientific method allows people to question the trustworthiness of the information sources and to deal with uncertainty, which ultimately helps to fight the spreading of misinformation (Awandare et al., 2020). Thus, scientific dissemination is not only a right, but also a duty (Lopez-Goñi, 2020).

Science and technology have become crucial tools to tackle the grand challenges of humanity. However, no matter how hard researchers work if their insights do not catch properly the attention to those who have the power to take real actions. The lack of a speaking ground language hinders communication and collaboration between scientists, diplomats, and policymakers. Furthermore, whereas the scientists complain about the low knowledge and interest in the science of the policymakers, the latter blame the researchers for not working on relevant projects and not supplying the information they needed immediately (Janse, 2008).

Latin America is a huge and highly diverse region, with important socioeconomic and cultural differences among countries. There is also a tremendous disparity in scientific production. But even rich Latin American nations do not produce a good level of science, as a result of the negative environment created by political leadership, rather than the lack of talent or creativity (Ciocca and Delgado, 2017). Despite this disadvantage, in countries like Brazil, Argentina, or Colombia, scientific journalism began as early as in the 19th century, when scientific and technological advances appeared within the pages of the first printed newspapers, even before there was a recognizable academic scientific community within the region (Vessuri, 1994; Fog, 2004; Nowak, 2008; Massarani, 2010). Science and natural history museums, as well as botanical gardens, have also a long history, existing in Brazil, Mexico, or Uruguay for more than 200 years (Vessuri, 1994; Massarani, 2015; Sánchez-Mora et al., 2015). However, it was not until the 1960s, when scientific communication gained relevance as part of a growing educational movement that sought to increase the scientific culture of the population, in several Latin American countries (Massarani et al., 2015).

A substantial body of literature identifies communication as one of the key skills for successful evidence-informed policymaking and to close the gap between the so-called “two communities” (Tseng, 2012; Akerlof et al., 2018; Topp et al., 2018; Yanovitzky and Weber, 2018; Zdunek et al., 2021). Langlois et al. (2019) found that adequate communication incentives and training of human resources were the main facilitators to embed research into policy, in eight Latin American and the Caribbean countries.

In this perspective, we aim to provide recommendations on how to bring Latin American policymakers to science through communication by combining insights from relevant stakeholders in the region with previous findings and from the personal opinions of the authors based on their own experience.

## Listening to the Stakeholders

There is a consensus that scientific knowledge does not reach decision-makers properly. About 78% of diplomats and policymakers as well as 89.8% of researchers and science journalists agreed with this statement, in an online survey distributed within Latin America, from September to November 2020 (Supplementary Methods).

**Figure 1:**
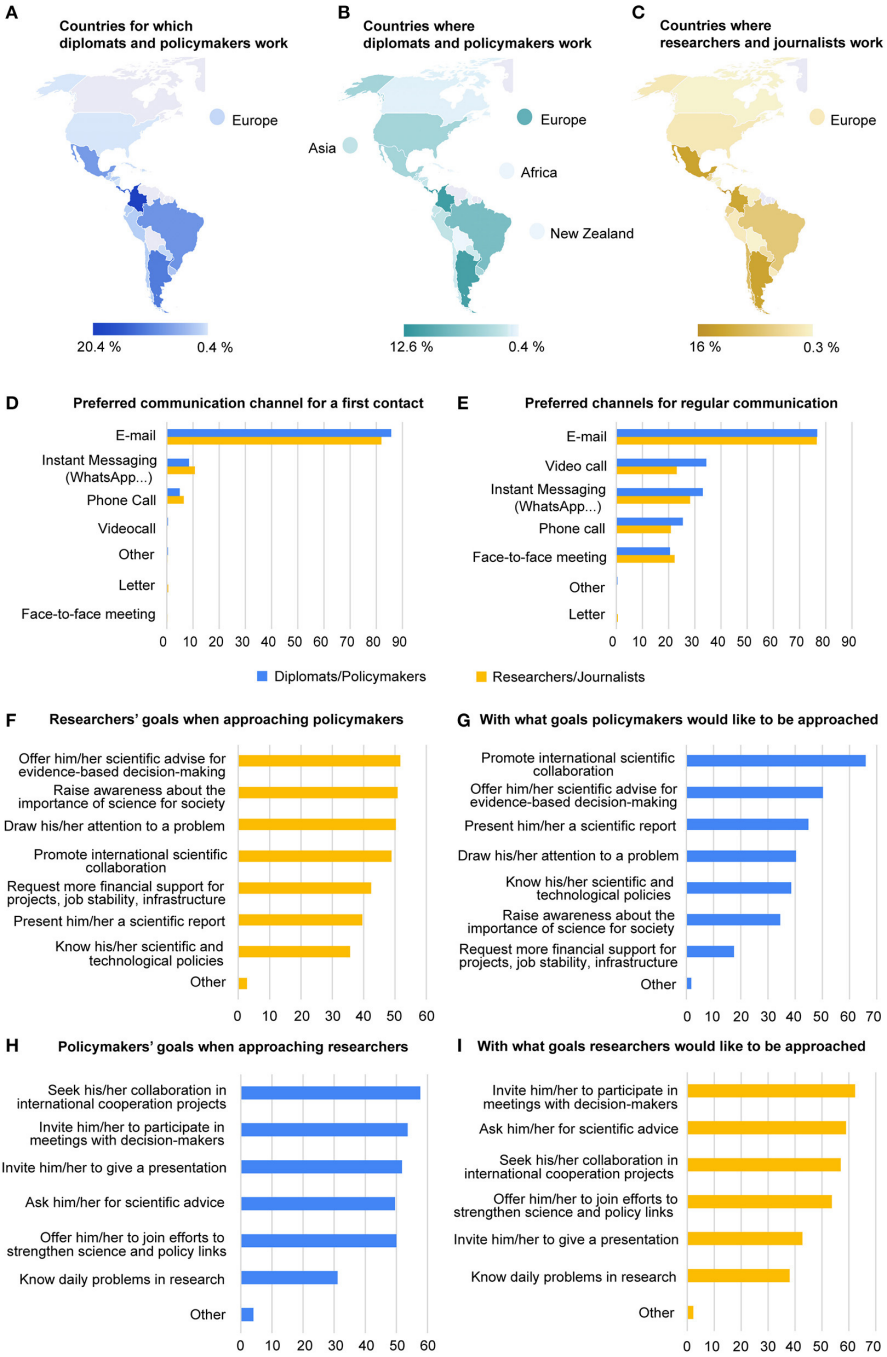
**(A)** Countries for which diplomats and policymakers work. **(B)** Countries where diplomats and policymakers carried out their professional activity. **(C)** Countries where researchers and journalists carried out their professional activity. **(D)** Preferred communication channels to stabilize a first contact according to diplomats and policymakers (blue), as well as researchers and science journalists (yellow). **(E)** Preferred channels for regular communication as reported by diplomats and policymakers (blue), as well as researchers and science journalists (yellow). **(F)** Goals of researchers and science journalists when communicating with diplomats and policymakers. **(G)** Reasons why diplomats and policymakers would like to be reached out by researchers and science journalists. **(H)** Goals of diplomats and policymakers when communicating with researchers and science journalists. **(I)** Reasons why researchers and science journalists would like to be reached out by diplomats and policymakers. All data are shown in percent.

The first questionnaire, designed for diplomats and policymakers got 225 participants, whereas the questionnaire of researchers and science journalists was answered by 362 people (Supplementary Tables 1–4 for demographic information of the respondents). More than 75% of diplomats and policymakers represented Colombia, Panama, Costa Rica, Argentina, Brazil, and Mexico (Figure 1A and Supplementary Table 5), whereas they were mostly working in Panama, Colombia, Argentina, Europe, Costa Rica, Brazil, Mexico, and Uruguay (Figure 1B and Supplementary Table 6). As far as researchers and science journalists are concerned, most of them were settled in Panama, Mexico, Argentina, Colombia, Chile, and Brazil (Figure 1C and Supplementary Table 7).

Although responses were obtained from almost all Latin American countries, we are aware that the sample does not represent the region homogeneously. But data on science communication efforts targeting Latin American policymakers are scarce. Thus, we prefer to listen to stakeholders in the region, even partially, to elaborate recommendations tailored to the real problems they face when communicating, instead of writing a theoretical assay based solely on the experiences of non-Latin American countries.

## Preparing to Reach Policymakers

Many scientists have a strong motivation to increase the impact of their work, and to engage with policymakers, although they might not know how to start. Among our questionnaire respondents, 58.6% of researchers and science journalists declare to interact with diplomats and policymakers, on a biannually (44.4%), monthly (29.5%), and weekly (12.6%) basis. Catching their attention or even getting an answer could be quite challenging, as experienced by ourselves when distributing our survey. However, correctly identifying the who, when, and how improves the chances of success (Cairney and Kwiatkowski, 2017; Topp et al., 2018).

Reaching policymakers who have science issues on their agenda is much easier than getting an answer from others devoted to other interests. In our research, we have found that many Latin American countries provide information regarding draft laws, initiatives, and commissions in which decision-makers take part, on their Parliamentary website. It might be worth visiting to identify the target of whom to communicate science.

A common practice within the policy is lobbying (Thomas and Klimovich, 2014). So, diplomats and policymakers can be quite suspicious when receiving a “cold call.” From our experience, being honest about goals and motivations increase the likeliness they trust and listen to you, as do recommendations. Talking to those around and establishing a valuable network of contacts can help close the gap between science communicators and decision-makers, too.

Timing is also important. While researchers usually work on long-term projects, time in policy is counted in months and is heavily impacted by electoral calendars. Therefore, we would not advice researchers to approach policymakers while campaigning. However, meeting at the beginning of a policy term, when priorities are being set, is quite effective (Safford and Brown, 2019).

Latin American researchers, science journalists, diplomats, and policymakers prefer email as an ice-breaking communication channel (Figure 1D). It works on regular communications too (Figure 1E). Personalized subjects and salutations are a must to get attention, while email addresses can be found on many Latin American official and governmental websites. Once the parties know each other, video calls, instant messaging, phone calls, and face-to-face meetings can reinforce communication (Figure 1E).

## Common Interests, but Different Priorities

It is often said that science and policy are far away from each other and that researchers and policymakers are strange bedfellows with little or no common interests (Lucente, 2017). However, it might not be entirely true. According to our survey results, researchers and policymakers may indeed share goals, but prioritize them differently. For instance, the main objectives of Latin American researchers when approaching policymakers include, “offering scientific knowledge for evidence-based decision-making,” “raising awareness about the importance of science in society,” and “drawing policymaker's attention to a problem” (Figure 1F). However, at first, the policymakers would like to be approached to “promote international scientific collaboration,” (Figure 1G) something researchers rank as their fourth priority.

Differences also arise the other way around. In the first instance, Latin American diplomats and policymakers would contact researchers to “seek their collaboration in international cooperation projects” (Figure 1H). Secondly, they would “invite researchers to participate in meeting with decision-makers,” which represents the main interest of researchers when reached by diplomats and policymakers (Figure 1I). Policymakers would also like to “invite researchers to give a presentation” (Figure 1H). However, researchers would prefer to be reached out to “ask them for scientific advice” (Figure 1I). Finally, the one and the other rank “decision-makers gain first-hand knowledge of daily problems in research” as the least important input. The low priority given by researchers to this issue has surprised the authors.

Nevertheless, it is worth noting that regardless of how both parties ranked the inputs, even the lowest rated got a significant percentage of attention. This, in our opinion, means that there are indeed common interests to start building a dialogue, even if it is not always easy or successful.

## Seeking Common Communication Channels

Most common difficulties faced by the Latin American researchers and science journalists when engaging with diplomats and policymakers are lack of interest or time, as well as scientific illiteracy and ignorance on the relevance of science to decision-making. Mistrust also pervades Latin American scientists who fear the misuse of their data to support political and economic interests. Although a scenario where every decision is based on evidence is quite unrealistic, because uncertainty is intrinsic to science that does not have every answer, there is a common demand among Latin American researchers to increase science influence in policy. To achieve this goal, many of them stated that science communication could be a facilitator.

**Figure 2:**
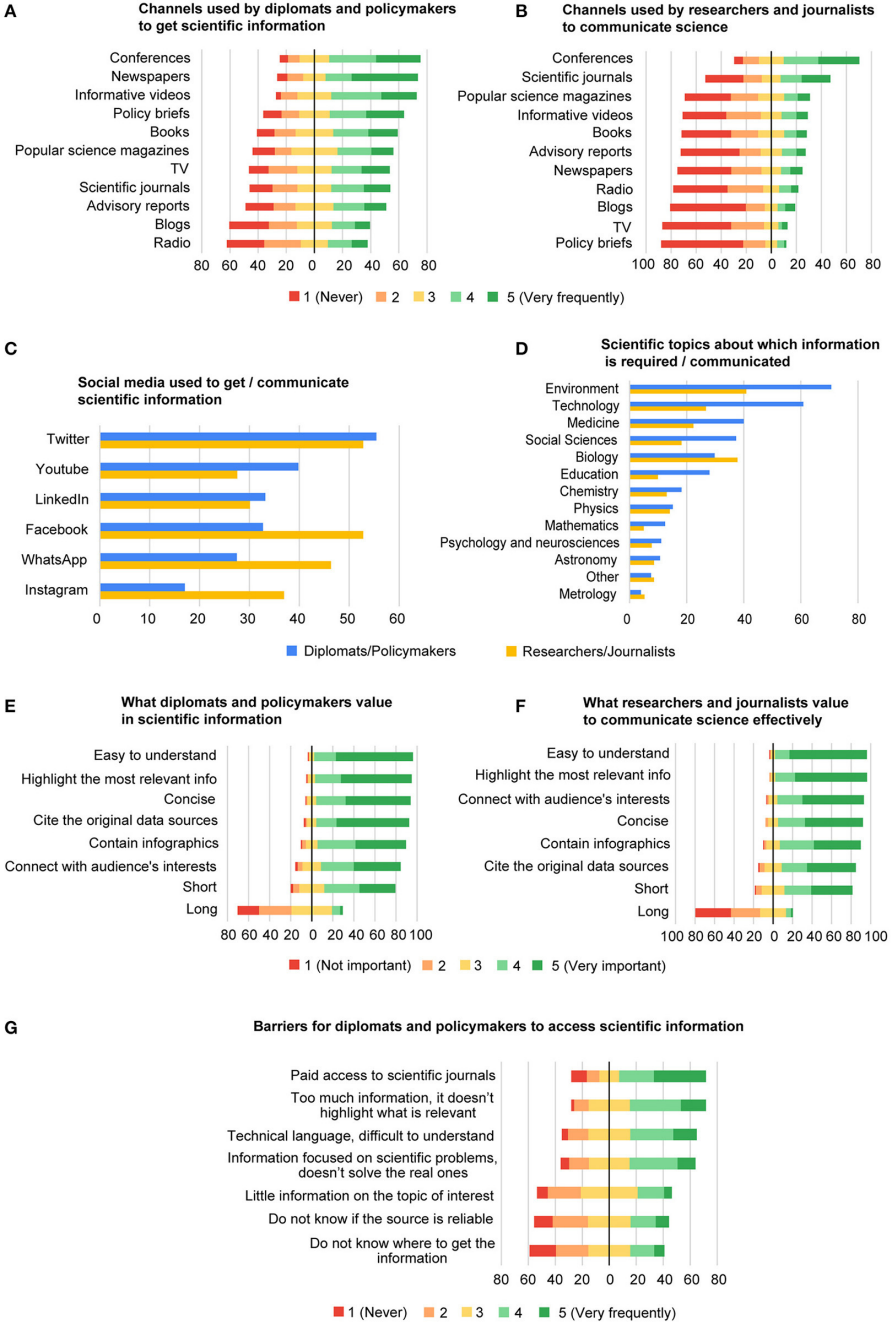
**(A)** Frequency at which diplomats and policymakers use various communication media to obtain information on scientific and technological advances, in their work. **(B)** Frequency at which researchers and science journalists communicate science on various communication media. **(C)** Preferred social media to get scientific information (diplomats and policymakers, blue) and to communicate science (researchers and science journalists, yellow). **(D)** Scientific topics about which diplomats and policymakers need information (blue) and researchers and science journalists focus on when communicating (yellow). **(E)** What diplomats and policymakers value more or less in science information. **(F)** Factors, the researchers and science journalists think are more or less important for effective science communication. **(G)** Frequency at which diplomats and policymakers face different barriers when trying to acquire scientific information. All data are shown in percent.

Significance and usefulness of scientific data are limited, if not shared. Thus, whether in peer-reviewed journals or scientific meetings, researchers spend most of their academic career in communicating their knowledge. However, policymakers are not always good at reading a scientific paper, because it is not their job (Streubel, 2018). Latin American diplomats and policymakers would rather attend conferences to get information about scientific and technological advances (Figure 2A). Conferences are also the preferred media of researchers and science journalists to communicate science (Figure 2B). Nevertheless, we advise on not giving a long talk, with the vocabulary and format of a scientific conference, before a busy decision-maker, since it will hardly have any impact and will probably end in failure.

Communicating to a non-scientific audience requires training, something that interviewed Latin American researchers claim to miss. This is reflected in their little use of generalist media as a speaker, in spite of recognizing that newspapers are one of the main sources of information used by decision-makers (Figures 2A,B). Informative videos also require some specific skills, but the reward is worth the effort. Images are a particularly efficient method of communicating information, which allow conveying of large amounts of data in a relatively short space of time (Pasquali, 2007). So, considering that diplomats and policymakers have busy and awkward lives (Docquier, 2017), the high marks they give to videos are not surprising (Figure 2A). Latin American researchers and science journalists also account for the usefulness of videos (Figure 2B). They not only make it easier to explain complex and tedious manuscripts (Darzentas et al., 2007), but also constitute an effective way to portray an accurate view of how science gets done, as well as some of the interesting places where scientists work (Pasquali, 2007). However, a document that specifically addresses the interests and the needs of policymakers is largely ignored by Latin American researchers and science journalists (Figure 2B). Policy briefs give concise, objective summaries of relevant scientific data, as well as recommendations, aimed to help readers decide what they should do (DeMarco and Tufts, 2014). We encourage the Latin American scientific community to communicate through this channel to bring research into policy.

## Who Is Tweeting?

Social media deserve a special mention, since they provide researchers with one of the most direct routes for sharing their work, as well as reaching practitioners and the general public. In fact, Latin American diplomats and policymakers make extensive use of social media to obtain information on scientific issues; especially, Twitter (Figure 2C). Communicating science through Twitter is a challenge, as it requires condensing complex messages into very little space. Still, together with Facebook, it is the most used social media by Latin American researchers and science journalists to spread their knowledge (Figure 2C).

Twitter is an ideal tool for broadcasting, but even more so for listening and discussing. Therefore, we encourage Latin American researchers and science journalists to take advantage of this social network to connect with people (e.g., diplomats and policymakers), beyond those who share their opinions and interests. That is, to avoid falling into the so-called “echo chamber” to increase the impact of their message. Furthermore, tweeted articles achieve higher citation rates, which suggest a wider reach also among scientist peers (Klar et al., 2020; Luc et al., 2021).

YouTube is the second most visited website globally and also the second most popular social network, with more than 1,500 million users, after Facebook (Fernández Bayo et al., 2019). Considering the fact that Latin American diplomats and policymakers use informative videos as scientific information sources, it comes as no surprise they rank YouTube as their second-most used social media (Figure 2C). What is quite shocking is that it is the least one used by Latin American researchers and science journalists. Certainly, in our opinion, it is an aspect to correct and improve.

There is also little agreement on the preferences of both parties with regard to the use of LinkedIn, Facebook, WhatsApp, and Instagram (Figure 2C). However, for us, it is worthy to take advantage of any opportunity to communicate. Thus, this should not discourage researchers from learning and adapting their message to each social network in order to engage with decision-makers.

## Bringing Science to Policymakers

To be effective, science communication should cover the needs of the audience (National Academies of Sciences, 2017). In Latin America, diplomats and policymakers request information about environmental issues (Figure 2D). Since the region faces many challenges, from forest fires that devastated much of the continent, to contamination of soil and water resources, or the vulnerability of Central America to natural disasters aggravated by climate change, the environment is also the main topic communicated by researchers and science journalists. Interestingly, when addressing this issue, Latin American researchers and policymakers can find a common ground language, quite easy. Noticing that, our survey shows that policymakers demand information on a wide variety of topics, which we see as an opportunity for researchers to advice policy.

And when doing so, translating research findings into simple, but not simpler, easy to understand language is a necessity for evidence communication (Gregrich, 2003). Highlighting the most relevant information, along with coming to the point, that is, being concise using few and adequate words, is what Latin American diplomats and policymakers value most in scientific information (Figure 2E). According to the personal experience of the Latin American researchers and science journalists, considering their audience interests when disseminating science is a key to ensure successful communication (Figure 2F). Interestingly, both parties are unanimous in rating what makes science communication effective (Figures 2E,F), which suggest that finding a common language to put research on use is indeed possible.

At this point, it seems evident that Latin American diplomats and policymakers are interested in science, or at least part of their community. However, they often face paywalls when trying to access original manuscripts. Moreover, to the excess of irrelevant information and the incomprehensibility of technical language, they add the difficulty of finding research that responds to policy questions and social concerns (Figure 2G). Therefore, although it is true that there is a difference at the educational level between researchers and policymakers (Supplementary Tables 8, 9), the latter are not illiterate. Hence, in our opinion, they might just need a bridge to the scientific community.

## Discussion and Conclusions

Successful communication of science serves as a prerequisite for the successful use of science in policy (Akerlof, 2018). In the last few decades, science communication has grown significantly in Latin America. Internet and social media have greatly contributed to it, but there is still a long way to go (Massarani, 2018). Most Latin American research institutions have a limited budget devoted to science communication activities. Barba et al. (2019) found that only 10% of their staff undertaking science communication activities were professionals and, of them, only 35.6% made it on a full-time basis. This is in accordance with our results, as 73.9% of researchers declare to be volunteers, and not always being paid for their dissemination. Moreover, in our survey, science journalists claim being an endangered species in the midst of a media crisis, aggravated by difficult economic times, where coverage of science is considered expendable and, if necessary, carried out by non-specialist reporters.

There is a common consensus among Latin American researchers, science journalists, diplomats, and policymakers that scientific knowledge does not adequately reach decision-makers. That gap relates primarily to what is sometimes described as the “two communities” problem, and the key differences in their practices, rules, expectations, incentives, and language (Gaudreau and Saner, 2014). No simple answer exists to deal with this problem, but communication is one of the most cited skills to address it (Cherney and Head, 2010; Leshner, 2012; Akerlof et al., 2018; Topp et al., 2018).

After interviewing fifteen US Congress members, Akerlof et al. (2018) identified complexity, evidence inconclusiveness, accessibility, presentation, and lack of data transparency as barriers to the use of science in policy. The review by Oliver and Cairney (2019) on 145 studies found that access to information, clarity, relevance, and reliability of findings limit the use of evidence in health policy. These results align with the most frequent impediments that Latin American policymakers report facing in obtaining scientific information (Figure 2G).

What Latin American diplomats and policymakers value most in scientific information is that it is easy to understand, followed by hitting the main points and being concise (Figure 2E). Accordingly, some authors indicate the use of specialized language and scientific jargon as the reason why academic research often misses the attention of the policymakers (Feldman et al., 2001; Hemsley-Brown, 2004). They also highlight the importance of the way information is presented and the usefulness of visual formats, such as infographics or videos, which also attract Latin American policymakers, based on their use of informative videos and YouTube.

There is a groundswell of opinion, and the authors share their vision, that advocates the need to understand the audience to communicate science effectively (Cairney and Kwiatkowski, 2017; Davidson, 2017). Latin American policymakers report receiving too much information and not knowing how to identify which information fits their needs. Researchers are responsible for the way they present their data to reduce the burden on policymakers and facilitate their implementation in the decision-making process. In this sense, researchers should make use of the power of telling stories, documented in gray literature, to facilitate the memorization of information. Davidson recommended the Smart Chart (Spitfire Strategies, 2017) as the first step to build a communication strategy, along with the Message Box Workbook to extract the most relevant parts of the scientific message.

Researchers should also understand the timing and the real-world policymaking, far from the rational and orderly scientific cycle, as well as the use of science for policy, to tailor their message. Researchers often hope for instrumental use, wherein science directly influences a policy (Tseng, 2012). When questioned about this topic, Latin American researchers bemoan political or symbolic uses, in which research is used to justify a position that has already been fixed. But not infrequently, the use of information may actually occur after the decision has been made. Here, the elaborative use can refine the already defined position, whereas the strategic use serves to reconfirm it (Akerlof, 2018). Researchers can also influence how policymakers think about problems or potential solutions (i.e., conceptual use) (Tseng, 2012).

After analyzing the responses of Latin American researchers and policymakers, we concluded that there is a general lack of knowledge of the other's world. We recommend bringing both parties together to discuss common issues to improve communication. Cross-training seems to facilitate collaboration by stabilizing mutual understanding of language and values (Gaudreau and Saner, 2014). Accordingly, we highlighted initiatives, such as the science, technology, policy (STeP) Fellowship Program held by the Inter-American Institute for Global Change Research, within the Latin American region, whose fellows engage first-hand with policymakers. We also encourage researchers who are concerned about improving their science communication skills to enroll in training programs (see Massarani et al., 2016 for an overview of the current postgraduate opportunities existing in Latin America).

Latin America has the potential to build bridges between science and policy. However, further research collecting testimonies, failures, and success stories in the region is needed to provide the best practices and guidance to improve communication between researchers and policymakers. In this perspective, we aimed to give a general, descriptive overview of the region that serves as the first step. But effective application and adoption of evidence-based approaches require identification of “what works for whom in what circumstances” (Cherney and Head, 2010). This makes it difficult to duplicate strictly between countries and highlights the need to study each region in particular.

## Data Availability Statement

The raw data supporting the conclusions of this article will be made available by the authors, without undue reservation upon request.

## Author Contributions

MP-S conceived the idea, designed the questionnaires, distributed them, analyzed the data, and wrote the initial manuscript. FACM approved the questionnaires, distributed them, supervised data analysis, and extensively revised the paper. All authors contributed to the article and approved the submitted version.

## Conflict of Interest

The authors declare that the research was conducted in the absence of any commercial or financial relationships that could be construed as a potential conflict of interest.
