# Supplementary material for: Bringing Policymakers to Science Through Communication: A Perspective From Latin America
Source: Front Res Metr Anal. 2021 Apr 26;6:654191. doi: 10.3389/frma.2021.654191 (PMC8107434; doi:10.3389/frma.2021.654191)
Supplement: Supplementary file 1 [file Data_Sheet_1.pdf]

Supplementary methods

Sample

Information was gathered for two months, from September to November 2020, by means of two online surveys. The first one was addressed to researchers and scientific journalists, whereas the second one to diplomats and policymakers, from or based in Latin America. Questionnaires were distributed by direct invitation to universities, research centers, science academies, science journalists networks, public agencies, diplomatic academies, embassies, parliamentary science committees, ministries and relevant individuals within Latin America. Authors also asked study subjects to recruit respondents from among their acquaintances (snowball sampling).

The survey

The two questionnaires integrated 20 closed-ended and 7 open-ended questions. The first part of the surveys asked for demographic data (gender, age, studies, current occupation, country of work), which allowed to classify the answers. The second part comprised information related to the purpose of the survey: use of the different information channels to get/communicate scientific information, scientific topics about which information is required/communicated, information about how stakeholders interact (expectations, goals, communication channels), science communication barriers/facilitators and recommendations based on their opinions/experience.

Once validated by Latin American practitioners in science communication and research-policy interface, the questionnaires were entered in the online system (Google forms) in Spanish, Portuguese and English and launched.

Quantitative results were statistically processed using standard descriptive methods (ranking, percent), and qualitative results were organized using affinity diagrams.

Supplementary table 1: Participants' gender

Diplomats and policymakers			Researchers and science journalists		
		%			%
**Male**	104	46,2	**Male**	183	50,6
**Female**	120	53,3	**Female**	178	49,2
**Prefer not to say**	1	0,4	**Prefer not to say**	1	0,3

Supplementary table 2: Participants' age

Diplomats and policymakers			Researchers and science journalists		
		%			%
**Under 30**	22	9,8	**Under 30**	27	7,5
**30-40**	90	40,0	**30-40**	106	29,3
**41-50**	56	24,9	**41-50**	89	24,6
**51-60**	36	16,0	**51-60**	77	21,3
**Over 60**	21	9,3	**Over 60**	63	17,4

Supplementary table 3: Diplomats and policymakers' current occupation

		%
**Civil servant (agency, ministry)**	92	40,9
**Diplomat (in embassies/consulates)**	87	38,7
**Advisor (to the diplomatic core, to politicians)**	21	9,3
**Politician (in a government, in a parliament)**	9	4,0
**Lecturer (Diplomatic Academy)**	5	2,2
**International Organization**	5	2,2
**Other**	4	1,8
**Student (Diplomatic Academy)**	2	0,9

Supplementary table 4: Researchers and science journalists' current occupation

		%
**Scientific researcher**	243	66,9
**Journalist**	36	9,9
**Communication officer at a University, Research Center or Public Agency**	25	6,9
**Other**	22	6,1
**Science disseminator**	20	5,5
**University lecturer**	16	4,4
**Student**	9	2,5
**NGO, Agency, Research Center Director**	6	1,7

Supplementary table 5: Countries for which diplomats and policymakers work

		%
**Colombia**	46	20,4
**Panama**	33	14,7
**Costa Rica**	30	13,3
**Argentina**	25	11,1
**Brazil**	19	8,4
**Mexico**	17	7,6
**Uruguay**	7	3,1
**Chile**	5	2,2
**Ecuador**	5	2,2
**El Salvador**	5	2,2
**Guatemala**	5	2,2
**Honduras**	5	2,2
**Peru**	5	2,2
**International Organization**	4	1,8
**Europe**	3	1,3
**Paraguay**	3	1,3
**Cuba**	2	0,9
**Dominican Republic**	2	0,9
**Nicaragua**	1	0,4
**Caribbean**	1	0,4
**USA**	1	0,4
**Undisclosed**	1	0,4

Supplementary table 6: Countries where diplomats and policymakers work

		%
**Panama**	30	12,6
**Argentina**	26	10,9
**Colombia**	27	11,3
**Europe**	24	10,1
**Costa Rica**	21	8,8
**Brazil**	17	7,1
**México**	10	4,2
**Uruguay**	10	4,2
**USA**	10	4,2
**Asia**	9	3,8
**Guatemala**	6	2,5
**Peru**	6	2,5
**El Salvador**	5	2,1
**Honduras**	5	2,1
**Nicaragua**	5	2,1
**Ecuador**	5	2,1
**Chile**	4	1,7
**Paraguay**	4	1,7
**Caribbean**	3	1,3
**Cuba**	2	0,8
**Canada**	2	0,8
**Undisclosed**	2	0,8
**Guyana**	1	0,4
**Bolivia**	1	0,4
**Dominican Republic**	1	0,4
**Africa**	1	0,4
**New Zealand**	1	0,4

Supplementary table 7: Countries where researchers and science journalists work

		%
**Panama**	59	16,0
**Mexico**	47	12,8
**Argentina**	46	12,5
**Colombia**	45	12,2
**Chile**	31	8,4
**Brazil**	25	6,8
**Costa Rica**	19	5,2
**Guatemala**	19	5,2
**Ecuador**	18	4,9
**Paraguay**	13	3,5
**USA**	7	1,9
**Europe**	8	2,2
**Uruguay**	6	1,6
**Peru**	6	1,6
**Venezuela**	4	1,1
**Cuba**	4	1,1
**Honduras**	2	0,5
**El Salvador**	2	0,5
**Trinidad and Tobago**	2	0,5
**Dominican Republic**	1	0,3
**Nicaragua**	1	0,3
**Bolivia**	1	0,3
**Canada**	1	0,3
**Global**	1	0,3

Supplementary table 8: Diplomats and policymakers' highest academic degree

		%
**Master's degree (e.g. MA, MS, MBA)**	136	60,4
**Bachelor's degree (e.g. BA, BS)**	44	19,6
**Doctoral/Professional degree (e.g. PhD, MD, JD)**	34	15,1
**Postgraduate**	8	3,6
**High School**	2	0,9
**None**	1	0,4

Supplementary table 9: Researchers and science journalists' highest academic degree

		%
**Doctoral/Professional degree (e.g. PhD, MD, JD)**	205	56,6
**Master's degree (e.g. MA, MS, MBA)**	98	27,1
**Bachelor's degree (e.g. BA, BS)**	48	13,3
**Postgraduate**	7	1,9
**High School**	3	0,8
**None**	1	0,3
